# Viral Coinfections

**DOI:** 10.3390/v14122645

**Published:** 2022-11-26

**Authors:** Yanting Du, Chen Wang, Ying Zhang

**Affiliations:** 1 Key Laboratory of Livestock Infectious Diseases, Ministry of Education, College of Animal Science and Veterinary Medicine, Shenyang Agricultural University, 120 Dongling Rd., Shenyang 110866, China; 2 Key Laboratory of Zoonosis, College of Animal Science and Veterinary Medicine, Shenyang Agricultural University, 120 Dongling Rd., Shenyang 110866, China

**Keywords:** viral coinfection, viral interaction, mechanism, research technique

## Abstract

In nature, viral coinfection is as widespread as viral infection alone. Viral coinfections often cause altered viral pathogenicity, disrupted host defense, and mixed-up clinical symptoms, all of which result in more difficult diagnosis and treatment of a disease. There are three major virus–virus interactions in coinfection cases: viral interference, viral synergy, and viral noninterference. We analyzed virus–virus interactions in both aspects of viruses and hosts and elucidated their possible mechanisms. Finally, we summarized the protocol of viral coinfection studies and key points in the process of virus separation and purification.

## 1. Introduction

In nature, it is common for multiple pathogens (viruses, bacteria, fungi, and protozoa) to infect the same host simultaneously or successively. This phenomenon is defined as coinfection [1]. Typically, coinfection complicates the symptoms and diagnosis of a disease. In this article, we only focus on viral coinfection in clinics while focusing on virus–virus interactions. 

A virus–virus interaction can be observed in five patterns: interference, synergy, noninterference, dependence assistance, and host–parasite relation. The most common virus–virus interaction in coinfection is interference, in which one virus competes to suppress the replication of another [2]. SARS-CoV-2 can extensively inhibit the replication of multiple respiratory viruses [3,4]. A persistent infection of the Old World arenavirus [5], influenza A virus (IAV) [6], or classical swine fever virus (CSFV) [7,8] eliminates the secondary viral infection; this is known as superinfection exclusion [6].

In contrast to interference, coinfection with certain viruses may enhance the replication of other viruses [9], which we define as synergy. For instance, West Nile virus (WNV) and IAV infection each enhance the replication of Culex flavivirus (CxFV) [10] and human parainfluenza virus type 2 (hPIV2) [9], respectively.

If coinfection has no effect on virus replication, it is defined as non-interference [11,12,13]. Noninterference is usually found between viruses with different tissue tropisms. In human or animal viral infections, we can often detect a “passenger virus” that does not cause any symptoms or disease. The relation between a “causative virus” and a “passenger virus” is independent.

Dependence assistance and host–parasite relations are two specific viral relationships. Viruses with an incomplete genome, such as adeno-associated virus, with defective interfering particles, cannot complicate a replication cycle by themselves; instead, they require the assistance of a “helper virus”, such as adeno virus, herpes virus, or another intact virus, in order to finish their life cycle. These represent dependence assistance in viral interactions [14,15,16].

A host–parasite relation exists between virophages and giant viruses [17]. Virophages, such as Sputnik, are parasites of Mimivirus and Mamavirus. Additionally, Sputnik cannot replicate in Acanthamoeba castellanii but grows rapidly in the giant virus producer found in amoebae coinfected with Mimivirus, and Sputnik growth impacts and reduces Mimivirus replication.

Interference, synergy, and noninterference interactions are commonly identified in clinical viral coinfection cases. In this review, we focus on these three viral interactions and summarize their outcomes, mechanisms, and relative studies.

## 2. Virus–Virus Interaction in Coinfections

**Figure 1:**
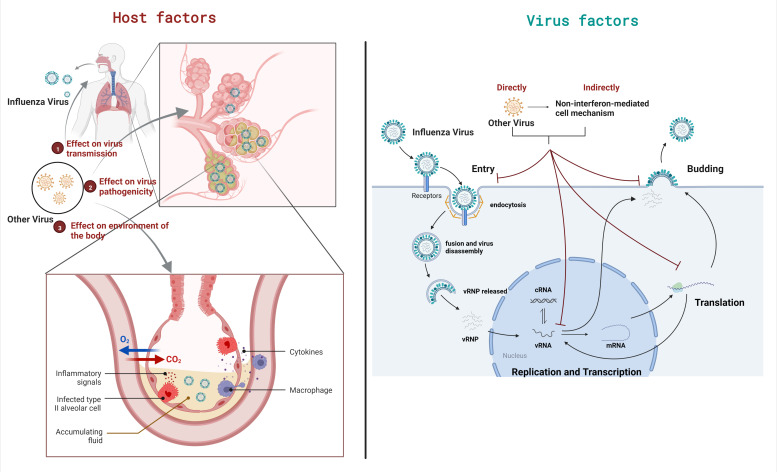
Two factors leading to the outcomes of viral coinfection. The outcomes of viral coinfection can be mainly attributed to two factors, namely virus factors and host factors. The figure takes IAV coinfection as an example. Host factors in coinfection change the environment of the body, thereby affecting the transmission and pathogenicity of viruses. Virus factors are coinfection changes that affect the intracellular environment and directly or indirectly affect the viral life cycle.

Interactions in viral coinfections are primarily caused by changes in virus replication cycles (virus factors) and the replication environment (host factors), as shown in Figure 1.

### 2.1. Viral Interference

**Figure 2:**
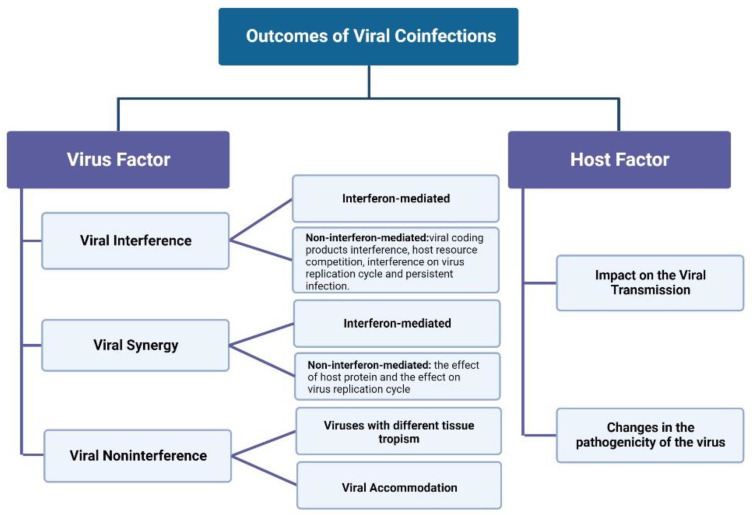
Outcomes of Viral Coinfections.

The causes of viral interference can be divided into two categories: interferon-mediated and non-interferon-mediated (Figure 2).

Interferon (IFN)-mediated innate immunity is the most common reason for viral interference [18,19]. In vivo studies of coinfection of IAV, respiratory syncytial virus (RSV), and rhinovirus (RV) show that IAV and RSV can interfere with RV replication through type I and type III IFN [20]. In clinical HCV and HIV coinfection, HIV-induced IFNα can reduce the level of HCV viremia [21]. Mouse hepatitis virus strain 1 (MHV-1) inhibits replication of IAV by upregulating IFN-β [22]. 

IFN induces multiple interferon-stimulating genes (ISGs) and activates multiple innate immunity signaling pathways [23,24,25,26,27,28,29,30,31,32,33]. GB virus C (GBV-C) inhibiting the proliferation of HIV is a typical IFN-mediated viral interference phenomenon. GBV-C promotes the activation of IFN-γ and downstream ISGs expression, as well as the activation/maturation of circulating pDC, which further increases IFN-γ [34]. Additionally, regarding coinfection of RV and IAV/pneumonia virus of mice (PVM), RV significantly inhibits the replication of IAV or PVM. RV induces an increase in Muc5ac gene expression, leading to an increase in IFN-β through the aromatic hydrocarbon receptor (AhR) signal transduction [35,36].

Non-interferon-mediated viral interference, also known as intrinsic interference, is the resistance of viral-infected cells to subsequent viral infections. This is particularly noticeable in foot-and-mouth disease virus (FMDV) coinfection cases, in which the attenuated A24 Cruzeiro strain interferes with the proliferation of homologous and heterologous strains [37]. Another typical intrinsic interference is in the case of Sindbis virus coinfection in viral infected vertebrate cells; the first virus translates non-structural genes to establish homologous exclusion and the genome of the second virus translates only without replication [38,39]. Adam et al. conducted further research based on these findings, and they found that a unipartite non-structural precursor called P123 is necessary to produce viral negative-strand RNA templates. The P123 of the latter virus is rapidly cleaved by the protease of the former virus, resulting in the latter virus being unable to synthesize the negative strand. This explains the phenomenon of intrinsic interference, at least to some extent [40].

The intrinsic interference between unrelated viruses can be found in the case of Newcastle disease virus (NDV) coinfection. Rubella virus can induce an interference state in infected host cells to avoid infection of NDV [41]. There is competition between the coinfected viruses for metabolites, replication sites [42], or a host’s viral replication-required proteins [12,40,43,44,45,46,47,48,49,50,51,52,53,54]. There are some host proteins that play a key role in the life cycle of various viruses, such as tetraspanins. Tetraspanins are transmembrane glycoproteins that are associated with the pathogenesis of non-enveloped viruses (human papillomavirus [HPV]) and enveloped viruses (HIV, Zika virus, IAV and coronavirus) [55]. When coinfections occur among these viruses, tetraspanins serve as the main host protein being explored.

In addition to the contest for host proteins, there are several other interference mediators, including defective interfering particles (DI particles) [56], RNA interferences (RNAi) [57,58,59,60,61], trans-acting viral proteins [62,63,64], and non-specific dsRNAs [65,66]. 

Virus interference can occur at each step of the virus-replication process, including virus attachment [67,68,69,70,71,72,73,74,75,76,77,78] and entry [54,79,80,81,82], viral genome replication [40,54,83,84,85,86,87,88], viral protein translation and assembly, and progeny virus budding [89]. At the stage of viral attachment, simian immunodeficiency virus (SIV) can significantly inhibit the expression of CD4 glycoprotein on the cell surface, which causes cell resistance to HIV-1 superinfection [78]. At the stage of entry, vesicular stomatitis virus (VSV) inhibits the formation rate of endocytic vesicles and reduces the internalization rate of receptor-binding ligands in order to restrain other viruses from taking over the coated pits [81]. In the viral gene-replication step, the expression of the Borna disease virus (BDV) P, N or X protein makes human cells resistant to superinfection with BDV by selectively blocking the polymerase activity of viruses [84]. In the viral protein translation step, the coinfection of VSV and IAV inhibits the translation of IAV mRNA, which is related to the inhibition of protein synthesis after VSV infection [90]. In the viral assembly and budding stages, Alphabaculovirus-induced actin recombination blocks the assembly and budding of other viruses [89]. Inhibition could happen at multiple steps, as Semliki Forest virus (SFV) infection inhibits the attachment, entry, and budding of subsequent viruses [54].

Viral interference is also often found in persistent infections. Unlike acute infections, in which virus particles are eventually cleared by the immune system or host, viruses stay in infected cells for a long time in persistent infections [1]. Viruses in persistent infections usually reduce their replication level [91,92,93,94,95,96,97,98,99] to keep the infected cell alive. Therefore, the virus in a persistent infection state can resist the influence of other viruses and exist in infected cells for a long time. A good example is the persistent infection of mosquitos by densovirus (DNV) [100]. DNV-infected cells are resistant to dengue virus (DENV) attack, and no CPE appears in these cases [101,102]. Studies on flock house virus (FHV) have shown that host and viral factors are involved in maintaining viral persistence [103,104,105]. Regarding the establishment of persistent infection in vitro, mutations in the viral genome begin to accumulate after several continuous passages [103], indicating that the cellular environment, rather than the virus itself, is essential for the establishment of sustained infection. The continuous replication of viruses could be accomplished by blocking the RNAi response of infected cells. Goic et al. reported that the persistence of FHV in *Drosophila melanogaster* could be accomplished by regulating RNAi and reverse transcriptase activity [106]. Fragments of different RNA viruses are reversely transcribed at early infection, which results in DNA forms embedded in the retrotransposon sequences. These virus-retrotransposon DNA chimeras trigger cellular RNAi mechanisms that inhibit viral replication. The inhibition of reverse transcriptase by FHV can hinder the emergence of chimeric DNA, thus closing the cell RNAi mechanism and making FHV persist in the cell.

### 2.2. Viral Synergy

The causes of viral synergy can be divided into two categories: interferon-mediated and non-interferon-mediated (as shown in Figure 2). Interferon-mediated viral promotion is primarily manifested as one virus causing host immunodeficiency; this, in turn, promotes the proliferation of the other viruses. In mouse L cells, coinfection with Vaccinia virus (VV) protects the VSV from IFN inhibition. This is related to the inhibition of IFN-induced dsRNA-dependent protein kinase activity by VV [107]. In coinfections of Hepatitis B virus (HBV) and Hepatitis C virus (HCV), the reduced liver IFN response after HCV clearance can cause HBV reactivation [108]. Another good example of this mechanism is the coinfection between canine parvovirus type 2 (CPV-2) and canine circovirus (CCV); CCV inhibits the activation of the IFN-I promoter by inducing Rep protein expression, thus blocking the subsequent expression of ISGs to promote CPV-2 replication [109]. Additionally, in the coinfection of paramyxovirus 5 wild-type (SV5-WT) and SV5 P/V mutant (rSV5-P/V-CPI−), rSV5-WT can block IFN signaling by inhibiting IRF-3 translocation into the nucleus and degrading STAT1 [110], thus blocking host cytokines involved in antiviral response and those involved in IFN synthesis [111].

Non-interferon-mediated viral synergy could be related to the effect of the host protein or the replication of other viruses. Coinfection between Marek’s disease virus (MDV) and reticuloendotheliosis virus (REV) increases the replication of both viruses in cells [112]. Further studies have shown that host proteins such as IRF7, MX1, TIMP3, and AKT1 may be related to the synergy of MDV and REV. In the coinfection of the Avian leukosis virus subgroup J (ALV-J) and REV, host protein TRIM62 increases replication of the two viruses by regulating the actin cytoskeleton [113]. 

The effect of coinfection on virus replication cycles is more intuitive and effective. A study by Goto et al. found that hPIV2 infection enhances IAV replication [9] by promoting the fusion of the infected cell’s membrane. 

### 2.3. Viral Noninterference

Noninterference is usually found between viruses with different tissue tropisms. For example, influenza viruses mainly infect the upper respiratory tract and lower respiratory tract, and occasionally infect extrapulmonary tissues, such as the eyes and intestines [114]. HPV infection, however, is mainly distributed on the skin, mouth, nasal cavity and genitals [115]. The tissue tropisms of these two viruses have almost no intersection; therefore, when coinfections between IAV and HPV occur, we generally assume that their relationship is one of noninterference.

A host could be actively and continuously infected by multiple viruses without any obvious signs of disease. This is called viral accommodation. Viral accommodation is usually observed in arthropods [12] and shrimps [102,116,117]. There is little evidence that shrimps or other arthropods have an immune system [118], but exposure to inactivated virions or envelope proteins allows them to acquire short-term resistance to viral attacks [119,120]. In shrimp, viral diseases are the result of virus-induced apoptosis, which is not mediated by the immune system [102,121,122,123]. In viral accommodation, multiple viruses can exist independently and stably in the same cell, and the possibility of gene exchange between them depends on the similarity between the coinfected viruses.

## 3. Outcome of Viral Coinfections on Host

The outcomes of viral coinfection attributed to the host can be divided into two categories: effects on viral transmission and viral pathogenicity (as shown in Figure 2).

### 3.1. Effects on Virus Transmission

Inhibition and promotion of virus transmission can both be found in viral coinfection. Coinfection with DENV2 and DENV4 produces a competitive inhibitory effect that reduces the spread of the viruses [124,125,126]. Natural coinfections of RV and IAV occur frequently in humans. RV interferes with IAV transmission by reducing IAV aerosols [127]. Regarding the promotion of virus transmission, one study found that coinfections of CxFV and WNV promoted WNV transmission [10]. Coinfections of Chikungunya virus and Zika virus in mosquitoes leads to an enhancement in transmission of the Zika virus [128]. Therefore, in order to fully understand the effects of viral coinfection on virus transmission, further studies in natural populations are needed.

### 3.2. Effects on Viral Pathogenicity

Increased pathogenicity of viruses is another common result of viral coinfection. For example, FMDV does not generally kill adult sheep and goats [129]. However, when FMDV is coinfected with peste des petits ruminants virus (PPRV), the mortality rate increases to 50% [130]. The admission rate of coinfection in intensive care units was higher than that of single infection in human viral coinfection cases [131,132,133]. HBV and HCV coinfection causes more severe fibrosis and cirrhosis, as well as higher liver-related mortality, than single infection [134]. Coinfections of guinea pig reovirus and SARS-CoV cause rapid animal death in vivo [135]. Mice coinfected with Autochthonous Group 1 and 2 Brazilian VV showed more severe disease than mice infected with one virus alone [136].

However, not all viral coinfections will aggravate viral pathogenicity, and viral coinfection may not change or even alleviate the symptoms of a disease. Lanjuan Li et al. analyzed the impact of SARS-CoV-2 and IAV on the risk of disease severity in 9498 patients and found no significant association between SARS-CoV-2 and IAV coinfection mortality [137]. Additionally, Xiang et al. suggest that HBV infection does not increase the severity and outcome of COVID-19 [138].

Alleviation of symptoms is mainly reflected in the coinfection of respiratory viruses. RV can reduce the severity of IAV due to a faster reduction in the pulmonary inflammatory response and faster clearance of IAV [22]. Martinez-Roig, A et al. investigated coinfection of respiratory viruses in children and found that the number of viruses detected in nasopharyngeal aspirates was inversely proportional to the number of days of aerobic therapy and hospital stay [139]. 

Therefore, whether the virulence of a virus in coinfection changes seems to be related to the virus involved in the coinfection. 

## 4. Study of Viral Coinfection

**Figure 3:**
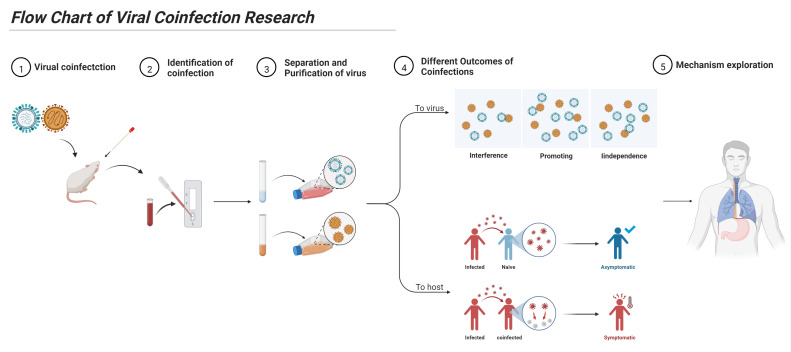
Flow chart of viral coinfection research. For the study of viral coinfection, it is necessary to correctly identify the occurrence of coinfections, establish a corresponding coinfection virus isolation and detection system, and determine the type of viral coinfection and its effect on a host. Finally, the mechanism of viral coinfection can be explored.

We summarized the study process of virus coinfection, as shown in Figure 3. The detailed methods are described below.

### 4.1. Identification

The diagnosis of coinfection and the separation of viruses in coinfection samples are the bottlenecks in studies of coinfection [140,141,142]. The identification of coinfections is traceable, and is often accompanied by increased or decreased clinical symptoms [143] and abnormal clinical symptoms (higher mortality, neurological symptoms, immunosuppression, etc.); these cannot be explained by single-pathogen infection [135,141]. Coinfections tend to have similar means of transmission (respiratory tract [144], vector [141], blood [140], etc.) and a similar host tropism. On the other hand, viruses in coinfections are often highly contagious and cross-represented in epidemic areas [142,145]. Therefore, suspected cases of coinfections can be identified from the above aspects.

The diagnosis of viruses is based on serological evidence and viral isolation. However, the sensitivity of serological methods is low and different viruses sometimes cause similar serological responses [146,147]. Virus isolation requires a suitable cell line or animal model, and the presence of multiple viruses may interfere with the replication of the target virus [148,149]. 

The development and application of PCR, qPCR and ELISA make the diagnosis of coinfection much simpler. PCR technology enhances the sensitivity of viral identification. However, PCR primers require the sequence information of the target virus, so PCR cannot identify novel viruses or unknown virus subspecies. The application of qPCR and ELISA technology makes up for the deficiency of PCR. By selecting genes or amino acid sites with high degeneracy, the versatility of detection is greatly improved and insufficient information regarding the unknown virus may be found. Novel coinfection identification methods are summarized as follows:

(i) The application of multiplex reverse-transcription quantitative real-time PCR (MRT-qPCR) [150,151,152], an improved version of qRT-PCR, makes coinfection detection more convenient and rapid. Its disadvantage is that building a new system takes a lot of time.
(ii) Application of digital droplet PCR (ddPCR) makes it possible to identify two highly similar viruses [153]. This method improves the accuracy and sensitivity of coinfection detection.
(iii) The transmission electron microscopy detection method of a gold nanoparticle gene probe also has applications in coinfection detection [154]. This method makes detection more convenient, which is conducive to clinical detection.
(iv) Fayyadh et al. used multicolor imaging with self-assembled quantum dot probes to image and successfully detect H1N1, H3N2, and H9N2 influenza viruses in coinfected cells [155]. This method provides a basis for in vitro detection of coinfection, which is more direct and easier to operate than traditional detection.
(v) Srisomwat et al. developed a point-of-care testing (POCT) device for HIV/HCV DNA detection [156]. Enhanced electroluminescence was observed in the presence of the target DNA by increasing proton conductivity [156]. This method has high specificity and a low cross-reaction for coinfection detection.

Although the identification of co-infections has been improved, the ability to detect target pathogens remains limited. The application of a next-generation sequencing (NGS) platform has improved virus diagnosis and the discovery of new viruses. NGS does not require prior sequence information about the target genome and can detect most potential genomes in clinical samples [157,158,159]. 

### 4.2. Viral Separation and Purification

Viral purification is extremely difficult in viral coinfection. In bacterial coinfection, different bacteria could be rapidly purified from a mixed culture by colony purification. On the other hand, multiple viruses cannot be easily purified directly from clinical samples. The isolation methods of viral coinfection mainly include CPE [130], organic solvent treatment (enveloped virus) [160], hemadsorption (separation of hemagglutination virus) [161], endpoint dilution assay [162], antibody (Ab) neutralization [163], acid/alkali treatment (PH-sensitive virus) [164], and reverse genetic system rescue [130]. The advantages and disadvantages of these methods are summarized below:

(i) The purification of viruses by CPE is a mainstream method for virus isolation in coinfection, but it requires the selection of suitable cell lines, where one virus can produce obvious CPE while the other virus does not produce obvious CPE. The disadvantage of this method is whether or not some traditional virus isolation cell lines are sensitive to another virus, and coinfection may affect the formation of CPE. At present, it is feasible to separate snakehead retrovirus (SnRV) from grouper nervous necrosis virus (GNNV) by SGF-1 [165]; FMDV from PPRV [130] or single serotype FMDV from multiple serotypes ofFMDV [142] by BHK21; IAV from respiratory viruses by suspended MDCK cells (MDCK-S) and adherent MDCK cells (MDCK-A) [166]; porcine epidemic diarrhea (PEDV) from porcine kobuvirus 1 (PKV) by Vero cells [157]; Hepatitis E virus (HEV) from porcine sapelovirus (PSV) by N1380 cells [167]; and porcine circovirus 2 (PCV2) from porcine parvovirus (PPV) by PK-15 [168].
(ii) An endpoint dilution assay is used to isolate two viruses with a highly similar host range/orientation but different replication rates. However, the separation success rate is usually low. It needs subsequent molecular-level detection and multi-generation blind passages for verification. Beperet et al. successfully isolated two different subtypes of alphabaculoviruses from coinfection samples by an endpoint dilution assay [162]. Dormitorio et al. successfully detected avian influenza virus (AIV) from suspicious allantoine fluid samples using this method [169].
(iii) The Ab neutralization method is suitable for different serotype viruses or two viruses with a distant genetic relationship. This method has a high success rate, but it needs to be verified by subsequent multi-generation blind passages. For the coinfection of multiple serotypes of the same virus, the serotype is generally determined first, and then the 2-dimensional microneutralization test (2D-MNT) corresponding to the serotype is carried out. Mahajan used 2D-MNT to isolate and purify multiple serotype viruses from coinfection samples of FMDV [142].

For coinfection, corresponding antibodies should be used, such as neutralizing PPRV in the coinfection of FMDV and PPRV [130], neutralizing NDV in coinfection of AIV and NDV [170], neutralizing CSFV from CSFV, and porcine astrovirus 5 (PAstV5) coinfection samples [171].

(iv) The organic solvent treatment method has certain limitations. Whether an organic solvent can kill one virus without affecting another virus needs to be verified. The choice of organic solvent is crucial. At present, it is feasible to remove PPRV with an organic solvent in coinfection of FMDV and PPRV [130]. The use of 5% H2O2 can completely inactivate the infectious laryngotracheitis virus, while the infectivity of NDV, infectious bronchitis virus, and AIV is reduced without being fully inactivated [172].
(v) Hemadsorption is suitable for virus isolation from non-hemagglutinating viruses. The integrity of this method for virus isolation is uncertain and the virus needs to be transferred to susceptible cell lines for amplification. At present, it is feasible to remove PPRV in coinfections of FMDV and PPRV [130]. Hemadsorption is useful for viruses such as IAV, parainfluenza virus, and mumps virus, which express their hemagglutinin proteins on the plasma membrane of infected cells [161].
(vi) Acid/alkali treatment is suitable for the separation of one PH-sensitive virus and another non-PH-sensitive virus. However, due to the difference in PH sensitivity of the isolated virus and the misdetection of molecular detection methods, this method has some notable limitations. Acidic environments (PH < 6.6) can effectively inhibit AIV replication [173]. The optimum survival range of the plague virus is from pH 6 to pH 11, while that of NDV is from pH 2 to pH 11 [174]. Thus, we can isolate viruses from coinfection samples by acid/alkali treatment.
(vii) Reverse genetic system rescues viruses. Some viruses have a mature reverse genetics system. We can isolate the complete genome fragments of the virus from the positive samples and then obtain complete or defective viruses. The disadvantage of this method is that constructing the system necessitates a considerable workload, and it is not suitable for the separation of two related viruses.

The successful isolation of viruses also depends on the cells used for virus purification. Sometimes, a single type of cell is not enough to isolate the virus [130,175,176]. Co-culture cells, i.e., a culture of multiple cell types together in a single layer, can solve the problem of isolating multiple viruses [161,177]. The mixture of MRC-5 and A549 cells can be used to detect cytomegalovirus (CMV), herpes simplex virus (HSV), and adenovirus in the same sample [177]. Mink lung and human adenocarcinoma cells (R-Mix) can be used for the rapid isolation of respiratory viruses (parainfluenza 1,2 and 3, influenza A and B, RSV, adenovirus, HSV, CMV and enterovirus) [178,179,180,181,182]. R-Mix cells also help in isolating highly pathogenic respiratory viruses, such as severe acute respiratory syndrome coronavirus (SARS-CoV). Another method currently being used is R-Mi Too cell line (composed of MDCK and A549 cells), which does not support SARS-CoV infection [183] but is more sensitive than R-Mix cells in the detection of influenza B virus and adenovirus [184]. Both R-Mix and R-Mix Too cells promote the growth of different influenza virus strains [185,186]. In addition, a mixture of MRC-5 and CV1 cells contributes to multiple detections of HSV-1, HSV-2, and varicella-zoster virus (VZV) [187,188]. Finally, Vero/BHK-21 co-culture cells could simultaneously isolate PPRV and FMDV [130]. However, the cost of co-culture cells is usually much higher than that of a single cell culture.

## 5. Conclusions

Viral coinfection is common but complicated. Studies of coinfection and virus–virus interactions represent an emerging field in virology. Due to coinfections, one virus infection could impact the outcome of another virus. A faster viral coinfection detection and virus separation system should be established for further study. In the future, viral coinfection studies will improve diagnoses, the development of vaccines, and antiviral therapy. 

To make it easier to study coinfections, we have outlined some of the literature on viral coinfections so that individuals can better select particular viruses of interest (Table 1).

## Figures and Tables

**Table 1:** Summary of viral coinfection *.

Coinfecting Viruses	Outcome	Method(s) of Detection	Method(s) of Purification	Cause Mechanisms	Effect on Host	Reference (Published Year)
HIV and HBV	NA	liver biopsies	NA	NA	Occurrence of complications and increased incidence of nonalcoholic fatty liver disease (NALFD)	[189] (2021)
COVID-19 and CoV 229E/OC43, AdV, HRV, FluA	Independence	MRT-qPCR	NA	NA	No obvious trend change	[190] (2021)
HPIV and HRV, RSV, AdV, HCoV, HboV, FluB, HMPV, FluA	NA	multiplex PCR	NA	NA	Alleviation of clinical symptoms in coinfection hosts	[191] (2019)
HBV and HCV	Noninterference (in vitro) coinfection interfered HBV (in vivo)	PCR, serologic profiles	NA	MiRNA 122 mediated by HCV core protein inhibits HBV replication.	A faster progression and high incidence of hepatocellular carcinoma	[192] (2018)
DENV, CHIKV, and ZIKV	NA	MRT-qPCR	NA	NA	Mean viraemia was significantly lower in coinfections compared to monoinfections. ZIKV- DENV coinfection did not significantly differ from reported ZIKV monoinfections. Coinfection by ZIKV–CHIKV could affect foetal death	[141] (2019)
FluA and hPIV2	coinfection enhanced FluA	Virus titration and Immunofluorescent staining		Cell fusion induced by hPIV2 infection promotes FluA replication.	NA	[9] (2016)
FluA and FluB	Noninterference	RT-PCR	Using Embryonating Chicken Eggs	NA	Patients presented typical influenza-like disease symptoms including fever > 39°C, myalgia, pharyngitis, and cough.	[193] (2013)
HBV, HCV, and HDV	Interference (HCV to HBV) Noninterference (HDV to HBV)	hepatitis B surface antigen loss rates	NA	NA	NA	[194] (2011)
RV and FluA	coinfection interfered FluA	Virus titration	NA	RV inhibits FluA replication by activating innate immune defense.	Reduced mortality in mice	[22] (2018)
SARS-CoV-2 and FluA	NA	Virus titration	NA	Coinfections caused severe lymphopenia in peripheral blood, resulting in reduced total IgG, neutralizing antibody titers, and CD4+ T cell responses against each virus.	The coinfection of SARS-CoV-2 with IAV enhanced disease severity.	[195] (2022)
Leprosy virus and HIV	Noninterference	clinical form and type of leprosy reaction	NA	HIV coinfected patients and patients with leprosy alone expressed similar levels of IL-1β and IL-6.	No change in tissue immunological behavior in patients coinfected with HIV and leprosy.	[196] (2017)
MDV and REV	Synergy	Confocal imaging, Western blotting, and qRT-PCR	Using the pfu and TCID50 methods	Two virus synergistic replication in vitro is related to innate immune pathway, Akt pathway, and cell adhesion and migration pathway.	Coinfection with Marek’s disease virus (MDV) and reticuloendotheliosis virus (REV) causes synergistic pathogenic effects and serious losses to the poultry industry.	[112] (2022)
DNV and CHIKV	Noninterference	RT-qPCR	NA	NA	The viruses could stably co-exist both in the cell lines and adult mosquitoes.	[100] (2010)
DNV and DENV	Interference (DNV to DENV)	Immunostaining for flow cytometry	Cell inoculated virus	NA	NA	[102] (2004)
DENV, DNV and JEV	Noninterference	Flow cytometry and IFA	Cell inoculated virus	NA	Triple co-infections of viruses can be easily established without signs of disease in C6/36 mosquito cells by sequential viral challenge followed by serial split passage of whole cells.	[197] (2010)
IBV and APV	Interference (IBV to APV)	RT-PCR	NA	NA	NA	[198] (2001)
IBV and NDV	Interference (IBV to NDV)	qRT-PCR	NA	NA	NA	[199] (2007)
HPAIV and NDV	Interference (NDV to HPAIV)	Virus titration	NA	This viral interference is titer dependent.	HPAIV replication was affected and an increase in survival was found in all coinfected groups when compared to the HPAIV single-inoculated group.	[148] (2016)
SINV and LACV	BHK cell: Enhancement(both SINV and LACV) C6/36 cell: coinfection don’t affect LACV; enhanced SINV	qRT-PCR	CPE	NA	NA	[149] (2014)
Sindbis Virus and other alphaviruses	Interference	Plaque assays	NA	This interference depends on a central role for the alphavirus trans-acting protease that processes the nonstructural proteins.	Mosquito cells persistently infected with Sindbis virus are broadly able to exclude other alphaviruses	[40] (1997)
WNV and CxFV	Noninterference (in vitro) Coinfection enhanced WNV (in vivo)	Plaque assays, qRT-PCR, and IFA	NA	The WNV titer in CxFV Izabal (+) C6/36 cells did not reach the maximum titer observed in CxFV Izabal (−) cells due to death of cells caused by CxFV Izabal.	NA	[10] (2010)
AIV and NDV	Interference	RT-PCR and serology	NA	NA	Coinfection with LPAIV had no impact on clinical signs; ducks coinfected with HPAIV survived for shorter duration.	[200] (2015)
HSV and VZV	Interference (superinfection exclusion, SE)	Laser confocal	Fluorescent virus rescue	The downregulation of heparan sulfate proteoglycan 2 (HSPG2) that alphaherpesvirus receptor may partially account for the exclusion.	NA	[201] (2014)
HMPV and HRSV	NA	ELISA and RT-PCR	NA	NA	Increased hospitalization rates	[144] (2005)
HCV and TTV	NA	PCR-HMA	NA	A generic method based upon PCR and heteroduplex mobility analysis (HMA) can be used to rapidly determine coinfection with two strains of the homologous virus.	NA	[202] (2000)
GaHV-1 and FWPV	NA	PCR	Using Embryonating Chicken Eggs and CPE	NA	NA	[203] (2010)
WSSV and IHHNV	NA	PCR and histopathology	NA	NA	Except for typical clinical symptoms of WSSV infection, coinfected shrimps did not have any other external deformities.	[204] (2014)
lvCIAV and iIBDV	Synergy	PCR, RT-PCR and ELISA	NA	LvCIAV infection attenuated subsequent iIBDV infection-induced T cell recruitment and subsequent B cell depletion in the bursa.	Without occurrence of clinical signs	[205] (2013)
Multiple coronaviruses	Noninterference	RT-PCR	NA	Bats are natural hosts of coronavirus and potential zoonotic sources of viral pathogens.	NA	[206] (2016)
HAdV, HEV, RSV and HRV	Noninterference	xTAG RVP Fast v2 and qRT-PCR	NA	NA	Lower frequency of lower respiratory tract infections, lower wheezing rates and higher hospitalization rates	[207] (2016)
HIV and FluA	Synergy	NA	NA	NA	Higher risk of influenza infection	[208] (2016)
PCV2 and CSFV	NA	proteomic profiling	NA	Mitochondrial dysfunction, nuclear factor erythroid 2-related factor 2 (Nrf2)-mediated oxidative stress response and apoptosis signaling pathways might be the specifical targets during PCV2-CSFV coinfection.	NA	[209] (2017)
PPRV and FMDV	Interference	qPT-PCR	Plaque assays, neutralization with antibodies and Viral RNA transfection	NA	NA	[130] (2016)
RSV and FluA	Interference	Virus titration and IFA	NA	FluA blocks the growth of RSV by competing with RSV for protein synthesis and selective budding.	NA	[210] (2000)
Two different FluA	Interference	Virus titration, RT-PCR and qRT-PCR	Plaque assays	H3N2 and H1N1 have different abilities to inhibit the replication and transmission of their respective drug-resistant virus mutants.	NA	[211] (2010)
PRRSV and SIV	Interference	IFA and qRT-PCR	Plaque assays and cell inoculated virus	PRRSV and SIV demonstrate additive effects on the expression of several types of virally induced transcripts.	NA	[212] (2014)
Two different VACV	Synergy (lung) Interference (spleen)	qPCR	NA	NA	NA	[136] (2018)
Two different WNV	Interference	Virus titration	NA	This interference depends on blocking the transmission of superinfecting virus.	NA	[213] (1969)
SLEV and WNV	Interference	qRT-PCR	NA	This interference depends on blocking the transmission of superinfecting virus.	NA	[214] (2009)
DENV1 and DENV3	Interference	IFA	NA	This interference depends on blocking the transmission of superinfecting virus.	NA	[215] (1982)

* Abbreviations: MRT-qPCR, multiplex reverse-transcription quantitative real-time PCR; HIV, Human immunodeficiency virus; HBV, Hepatitis B virus; HCV, Hepatitis C virus; HDV, Hepatitis D virus; HBoV human bocavirus; COVID-19, CoV 229E/OC43, SARS-CoV-2, HCoV, human coronavirus; AdV, human mastadenovirus A; HRV, Human rhinovirus B; FluA, Influenza A virus; FluB, influenza B virus; HPIV, human parainfluenza virus; RSV, respiratory syncytial virus; HMPV, human metapneumovirus; DENV, dengue virus; CHIKV, chikungunya virus; ZIKV, zika virus; hPIV2, human parainfluenza virus type 2; RV, Rhinovirus; MDV, Marek’s disease virus; REV, reticuloendotheliosis virus; DNV, densonucleosis viruses; CHIKV, Chikungunya fever virus; JEV, Japanese encephalitis virus; IBV, infectious bronchitis virus; APV, avian pneumovirus; NDV, Newcastle disease virus; AIV, Avian Influenza Virus; HPAIV, highly pathogenic AIV; SINV, Sindbis virus; LACV, La Crosse virus; WNV, West Nile virus; CxFV, Culex flavivirus; HSV, herpes simplex virus; VZV, Varicella-zoster virus; HRSV, human respiratory syncytial virus; TTV, Torque teno sus virus; GaHV-1, gallid herpesvirus 1; FWPV, fowlpox virus; WSSV, white spot syndrome virus; IHHNV, infectious hypodermal and hematopoietic necrosis virus; lvCIAV, low virulent T-lymphotropic chicken infectious anemia virus; iIBDV, intermediate B-lymphotropic infectious bursal disease virus; HAdV, human adenoviruses; HEV, human enterovirus; PCV2. porcine circovirus type 2; CSFV, classical swine fever virus; PPRV, peste des petits ruminants virus; FMDV, foot-and-mouth disease virus; PRRSV, porcine reproductive and respiratory syndrome virus; SIV, swine influenza virus; VACV, vaccinia virus; SLEV, St. Louis encephalitis virus.

## Data Availability

Not applicable.

## References

[1] Salas-Benito J.S., De Nova-Ocampo M. (2015). Viral Interference and Persistence in Mosquito-Borne Flaviviruses. J. Immunol. Res..

[2] Chu C.-J., Lee S.-D. (2008). Hepatitis B virus/hepatitis C virus coinfection: Epidemiology, clinical features, viral interactions and treatment. J. Gastroenterol. Hepatol..

[3] Halfmann P.J., Nakajima N., Sato Y., Takahashi K., Accola M., Chiba S., Fan S., Neumann G., Rehrauer W., Suzuki T. (2022). SARS-CoV-2 Interference of Influenza Virus Replication in Syrian Hamsters. J. Infect. Dis..

[4] Nowak M.D., Sordillo E.M., Gitman M.R., Paniz Mondolfi A.E. (2020). Coinfection in SARS-CoV-2 infected patients: Where are influenza virus and rhinovirus/enterovirus?. J. Med. Virol..

[5] Rojek J.M., Campbell K.P., Oldstone M.B., Kunz S. (2007). Old World arenavirus infection interferes with the expression of functional alpha-dystroglycan in the host cell. Mol. Biol. Cell.

[6] Huang I.C., Li W., Sui J., Marasco W., Choe H., Farzan M. (2008). Influenza A virus neuraminidase limits viral superinfection. J. Virol..

[7] Muñoz-González S., Perez-Simó M., Muñoz M., Bohorquez J.A., Rosell R., Summerfield A., Domingo M., Ruggli N., Ganges L. (2015). Efficacy of a live attenuated vaccine in classical swine fever virus postnatally persistently infected pigs. Vet. Res..

[8] Muñoz-González S., Pérez-Simó M., Colom-Cadena A., Cabezón O., Bohórquez J.A., Rosell R., Pérez L.J., Marco I., Lavín S., Domingo M. (2016). Classical Swine Fever Virus vs. Classical Swine Fever Virus: The Superinfection Exclusion Phenomenon in Experimentally Infected Wild Boar. PLoS ONE.

[9] Goto H., Ihira H., Morishita K., Tsuchiya M., Ohta K., Yumine N., Tsurudome M., Nishio M. (2016). Enhanced growth of influenza A virus by coinfection with human parainfluenza virus type 2. Med. Microbiol. Immunol..

[10] Kent R.J., Crabtree M.B., Miller B.R. (2010). Transmission of West Nile Virus by Culex quinquefasciatus Say Infected with Culex Flavivirus Izabal. PLoS Negl. Trop. Dis..

[11] Bellecave P., Gouttenoire J., Gajer M., Brass V., Koutsoudakis G., Blum H.E., Bartenschlager R., Nassal M., Moradpour D. (2009). Hepatitis B and C virus coinfection: A novel model system reveals the absence of direct viral interference. Hepatology.

[12] Kanthong N., Khemnu N., Sriurairatana S., Pattanakitsakul S.-N., Malasit P., Flegel T.W. (2008). Mosquito cells accommodate balanced, persistent co-infections with a densovirus and Dengue virus. Dev. Comp. Immunol..

[13] López-Vázquez C., Alonso M.C., Dopazo C.P., Bandín I. (2017). In vivo study of viral haemorrhagic septicaemia virus and infectious pancreatic necrosis virus coexistence in Senegalese sole (Solea senegalensis). J. Fish Dis..

[14] Gnanasekaran P., Chakraborty S. (2018). Biology of viral satellites and their role in pathogenesis. Curr. Opin. Virol..

[15] King A.M.Q., Adams M.J., Carstens E.B., Lefkowitz E.J. (2012). The Subviral Agents. Virus Taxonomy.

[16] King A.M.Q., Adams M.J., Carstens E.B., Lefkowitz E.J. (2012). Genus-Umbravirus. Virus Taxonomy.

[17] La Scola B., Desnues C., Pagnier I., Robert C., Barrassi L., Fournous G., Merchat M., Suzan-Monti M., Forterre P., Koonin E. (2008). The virophage as a unique parasite of the giant mimivirus. Nature.

[18] Haller O. (2015). Jean Lindenmann: From viral interference to interferon and beyond (1924–2015). J. Interferon Cytokine Res..

[19] Dianzani F. (1975). Viral interference and interferon. Ric. Clin. Lab..

[20] Essaidi-Laziosi M., Geiser J., Huang S., Constant S., Kaiser L., Tapparel C. (2020). Interferon-Dependent and Respiratory Virus-Specific Interference in Dual Infections of Airway Epithelia. Sci. Rep..

[21] Cribier B., Schmitt C., Rey D., Lang J.M., Kirn A., Stoll-Keller F. (1996). Role of endogenous interferon in hepatitis C virus (HCV) infection and in coinfection by HIV and HCV. Res. Virol..

[22] Gonzalez A.J., Ijezie E.C., Balemba O.B., Miura T.A. (2018). Attenuation of Influenza A Virus Disease Severity by Viral Coinfection in a Mouse Model. J. Virol..

[23] Wang X., Hinson E.R., Cresswell P. (2007). The interferon-inducible protein viperin inhibits influenza virus release by perturbing lipid rafts. Cell Host Microbe.

[24] Tan K.S., Olfat F., Phoon M.C., Hsu J.P., Howe J.L.C., Seet J.E., Chin K.C., Chow V.T.K. (2012). In vivo and in vitro studies on the antiviral activities of viperin against influenza H1N1 virus infection. J. Gen. Virol..

[25] Seo J.Y., Yaneva R., Cresswell P. (2011). Viperin: A multifunctional, interferon-inducible protein that regulates virus replication. Cell Host Microbe.

[26] Neil S.J. (2013). The antiviral activities of tetherin. Curr. Top. Microbiol. Immunol..

[27] Lenschow D.J. (2010). Antiviral Properties of ISG15. Viruses.

[28] Moldovan J.B., Moran J.V. (2015). The Zinc-Finger Antiviral Protein ZAP Inhibits LINE and Alu Retrotransposition. PLoS Genet..

[29] Carthagena L., Parise M.C., Ringeard M., Chelbi-Alix M.K., Hazan U., Nisole S. (2008). Implication of TRIM alpha and TRIMCyp in interferon-induced anti-retroviral restriction activities. Retrovirology.

[30] Chen K., Huang J., Zhang C., Huang S., Nunnari G., Wang F.X., Tong X., Gao L., Nikisher K., Zhang H. (2006). Alpha interferon potently enhances the anti-human immunodeficiency virus type 1 activity of APOBEC3G in resting primary CD4 T cells. J. Virol..

[31] Birdwell L.D., Zalinger Z.B., Li Y., Wright P.W., Elliott R., Rose K.M., Silverman R.H., Weiss S.R. (2016). Activation of RNase L by Murine Coronavirus in Myeloid Cells Is Dependent on Basal Oas Gene Expression and Independent of Virus-Induced Interferon. J. Virol..

[32] Nakayama M., Nagata K., Kato A., Ishihama A. (1991). Interferon-inducible mouse Mx1 protein that confers resistance to influenza virus is GTPase. J. Biol. Chem..

[33] Streitenfeld H., Boyd A., Fazakerley J.K., Bridgen A., Elliott R.M., Weber F. (2003). Activation of PKR by Bunyamwera virus is independent of the viral interferon antagonist NSs. J. Virol..

[34] Lalle E., Sacchi A., Abbate I., Vitale A., Martini F., D’Offizi G., Antonucci G., Castilletti C., Poccia F., Capobianchi M.R. (2008). Activation of interferon response genes and of plasmacytoid dendritic cells in HIV-1 positive subjects with GB virus C co-infection. Int. J. Immunopathol. Pharmacol..

[35] Liu Y., Lv J., Liu J., Li M., Xie J., Lv Q., Deng W., Zhou N., Zhou Y., Song J. (2020). Mucus production stimulated by IFN-AhR signaling triggers hypoxia of COVID-19. Cell Res..

[36] Leuven J.T.V., Gonzalez A.J., Ijezie E.C., Wixom A.Q., Clary J.L., Naranjo M.N., Ridenhour B.J., Miller C.R., Miura T.A. (2021). Rhinovirus Reduces the Severity of Subsequent Respiratory Viral Infections by Interferon-Dependent and -Independent Mechanisms. mSphere.

[37] Polacino P., Kaplan G., Palma E.L. (1985). Homologous interference by a foot-and-mouth disease virus strain attenuated for cattle. Arch. Virol..

[38] Adams R.H., Brown D.T. (1985). BHK cells expressing Sindbis virus-induced homologous interference allow the translation of nonstructural genes of superinfecting virus. J. Virol..

[39] Igarashi A., Koo R., Stollar V. (1977). Evolution and properties of Aedes albopictus cell cultures persistently infected with sindbis virus. Virology.

[40] Karpf A.R., Lenches E., Strauss E.G., Strauss J.H., Brown D.T. (1997). Superinfection exclusion of alphaviruses in three mosquito cell lines persistently infected with Sindbis virus. J. Virol..

[41] Marcus P.I., Carver D.H. (1967). Intrinsic interference: A new type of viral interference. J. Virol..

[42] Zebovitz E., Brown A. (1968). Interference among group A arboviruses. J. Virol..

[43] Brinton M.A. (2001). Host factors involved in West Nile virus replication. Ann. N. Y. Acad. Sci..

[44] Riis B., Rattan S.I., Clark B.F., Merrick W.C. (1990). Eukaryotic protein elongation factors. Trends Biochem. Sci..

[45] Li D., Wei T., Abbott C.M., Harrich D. (2013). The unexpected roles of eukaryotic translation elongation factors in RNA virus replication and pathogenesis. Microbiol. Mol. Biol. Rev. MMBR.

[46] Van Der Kelen K., Beyaert R., Inzé D., De Veylder L. (2009). Translational control of eukaryotic gene expression. Crit. Rev. Biochem. Mol. Biol..

[47] De Nova-Ocampo M., Villegas-Sepúlveda N., del Angel R.M. (2002). Translation elongation factor-1alpha, La, and PTB interact with the 3’ untranslated region of dengue 4 virus RNA. Virology.

[48] Davis W.G., Blackwell J.L., Shi P.Y., Brinton M.A. (2007). Interaction between the cellular protein eEF1A and the 3’-terminal stem-loop of West Nile virus genomic RNA facilitates viral minus-strand RNA synthesis. J. Virol..

[49] Blackwell J.L., Brinton M.A. (1997). Translation elongation factor-1 alpha interacts with the 3’ stem-loop region of West Nile virus genomic RNA. J. Virol..

[50] Yocupicio-Monroy M., Padmanabhan R., Medina F., del Angel R.M. (2007). Mosquito La protein binds to the 3’ untranslated region of the positive and negative polarity dengue virus RNAs and relocates to the cytoplasm of infected cells. Virology.

[51] García-Montalvo B.M., Medina F., del Angel R.M. (2004). La protein binds to NS5 and NS3 and to the 5’ and 3’ ends of Dengue 4 virus RNA. Virus Res..

[52] Gomila R.C., Martin G.W., Gehrke L. (2011). NF90 binds the dengue virus RNA 3’ terminus and is a positive regulator of dengue virus replication. PLoS ONE.

[53] Kenney J.L., Solberg O.D., Langevin S.A., Brault A.C. (2014). Characterization of a novel insect-specific flavivirus from Brazil: Potential for inhibition of infection of arthropod cells with medically important flaviviruses. J. Gen. Virol..

[54] Singh I.R., Suomalainen M., Varadarajan S., Garoff H., Helenius A. (1997). Multiple mechanisms for the inhibition of entry and uncoating of superinfecting Semliki Forest virus. Virology.

[55] New C., Lee Z.-Y., Tan K.S., Wong A.H., Wang D.Y., Tran T. (2021). Tetraspanins: Host Factors in Viral Infections. Int. J. Mol. Sci..

[56] Kim G.N., Kang C.Y. (2005). Utilization of homotypic and heterotypic proteins of vesicular stomatitis virus by defective interfering particle genomes for RNA replication and virion assembly: Implications for the mechanism of homologous viral interference. J. Virol..

[57] Chotkowski H.L., Ciota A.T., Jia Y., Puig-Basagoiti F., Kramer L.D., Shi P.Y., Glaser R.L. (2008). West Nile virus infection of Drosophila melanogaster induces a protective RNAi response. Virology.

[58] Sánchez-Vargas I., Scott J.C., Poole-Smith B.K., Franz A.W., Barbosa-Solomieu V., Wilusz J., Olson K.E., Blair C.D. (2009). Dengue virus type 2 infections of Aedes aegypti are modulated by the mosquito’s RNA interference pathway. PLoS Pathog..

[59] Sim S., Jupatanakul N., Ramirez J.L., Kang S., Romero-Vivas C.M., Mohammed H., Dimopoulos G. (2013). Transcriptomic profiling of diverse Aedes aegypti strains reveals increased basal-level immune activation in dengue virus-refractory populations and identifies novel virus-vector molecular interactions. PLoS Negl. Trop. Dis..

[60] Wu X., Hong H., Yue J., Wu Y., Li X., Jiang L., Li L., Li Q., Gao G., Yang X. (2010). Inhibitory effect of small interfering RNA on dengue virus replication in mosquito cells. Virol. J..

[61] Pijlman G.P. (2014). Flavivirus RNAi suppression: Decoding non-coding RNA. Curr. Opin. Virol..

[62] Lemm J.A., Rice C.M. (1993). Roles of nonstructural polyproteins and cleavage products in regulating Sindbis virus RNA replication and transcription. J. Virol..

[63] Lemm J.A., Rümenapf T., Strauss E.G., Strauss J.H., Rice C.M. (1994). Polypeptide requirements for assembly of functional Sindbis virus replication complexes: A model for the temporal regulation of minus- and plus-strand RNA synthesis. EMBO J..

[64] Shirako Y., Strauss J.H. (1994). Regulation of Sindbis virus RNA replication: Uncleaved P123 and nsP4 function in minus-strand RNA synthesis, whereas cleaved products from P123 are required for efficient plus-strand RNA synthesis. J. Virol..

[65] Nunes F.M., Aleixo A.C., Barchuk A.R., Bomtorin A.D., Grozinger C.M., Simões Z.L. (2013). Non-Target Effects of Green Fluorescent Protein (GFP)-Derived Double-Stranded RNA (dsRNA-GFP) Used in Honey Bee RNA Interference (RNAi) Assays. Insects.

[66] Flenniken M.L., Andino R. (2013). Non-Specific dsRNA-Mediated Antiviral Response in the Honey Bee. PLoS ONE.

[67] Steck F.T., Rubin H. (1966). The mechanism of interference between an avian leukosis virus and Rous sarcoma virus. II. Early steps of infection by RSV of cells under conditions of interference. Virology.

[68] Le Guern M., Levy J.A. (1992). Human immunodeficiency virus (HIV) type 1 can superinfect HIV-2-infected cells: Pseudotype virions produced with expanded cellular host range. Proc. Natl. Acad. Sci. USA.

[69] Michel N., Allespach I., Venzke S., Fackler O.T., Keppler O.T. (2005). The Nef protein of human immunodeficiency virus establishes superinfection immunity by a dual strategy to downregulate cell-surface CCR5 and CD4. Curr. Biol. CB.

[70] Hrecka K., Swigut T., Schindler M., Kirchhoff F., Skowronski J. (2005). Nef proteins from diverse groups of primate lentiviruses downmodulate CXCR4 to inhibit migration to the chemokine stromal derived factor 1. J. Virol..

[71] Geleziunas R., Bour S., Wainberg M.A. (1994). Cell surface down-modulation of CD4 after infection by HIV-1. FASEB J..

[72] Willey R.L., Maldarelli F., Martin M.A., Strebel K. (1992). Human immunodeficiency virus type 1 Vpu protein induces rapid degradation of CD4. J. Virol..

[73] Breiner K.M., Schaller H., Knolle P.A. (2001). Endothelial cell-mediated uptake of a hepatitis B virus: A new concept of liver targeting of hepatotropic microorganisms. Hepatology.

[74] Nethe M., Berkhout B., van der Kuyl A.C. (2005). Retroviral superinfection resistance. Retrovirology.

[75] Schneider-Schaulies J., Schnorr J.J., Brinckmann U., Dunster L.M., Baczko K., Liebert U.G., Schneider-Schaulies S., ter Meulen V. (1995). Receptor usage and differential downregulation of CD46 by measles virus wild-type and vaccine strains. Proc. Natl. Acad. Sci. USA.

[76] Walters K.A., Joyce M.A., Addison W.R., Fischer K.P., Tyrrell D.L. (2004). Superinfection exclusion in duck hepatitis B virus infection is mediated by the large surface antigen. J. Virol..

[77] Bratt M.A., Rubin H. (1968). Specific interference among strains of Newcastle disease virus. II. Comparison of interference by active and inactive virus. Virology.

[78] Benson R.E., Sanfridson A., Ottinger J.S., Doyle C., Cullen B.R. (1993). Downregulation of cell-surface CD4 expression by simian immunodeficiency virus Nef prevents viral super infection. J. Exp. Med..

[79] Rikkonen M., Peränen J., Kääriäinen L. (1992). Nuclear and nucleolar targeting signals of Semliki Forest virus nonstructural protein nsP2. Virology.

[80] Ranki M., Ulmanen I., Kääriäinen L. (1979). Semliki Forest virus-specific nonstructural protein is associated with ribosomes. FEBS Lett..

[81] Simon K.O., Cardamone J.J., Whitaker-Dowling P.A., Youngner J.S., Widnell C.C. (1990). Cellular mechanisms in the superinfection exclusion of vesicular stomatitis virus. Virology.

[82] Whitaker-Dowling P., Youngner J.S., Widnell C.C., Wilcox D.K. (1983). Superinfection exclusion by vesicular stomatitis virus. Virology.

[83] Zou G., Zhang B., Lim P.Y., Yuan Z., Bernard K.A., Shi P.Y. (2009). Exclusion of West Nile virus superinfection through RNA replication. J. Virol..

[84] Geib T., Sauder C., Venturelli S., Hässler C., Staeheli P., Schwemmle M. (2003). Selective virus resistance conferred by expression of Borna disease virus nucleocapsid components. J. Virol..

[85] Lohmann V., Hoffmann S., Herian U., Penin F., Bartenschlager R. (2003). Viral and cellular determinants of hepatitis C virus RNA replication in cell culture. J. Virol..

[86] Schaller T., Appel N., Koutsoudakis G., Kallis S., Lohmann V., Pietschmann T., Bartenschlager R. (2007). Analysis of hepatitis C virus superinfection exclusion by using novel fluorochrome gene-tagged viral genomes. J. Virol..

[87] Claus C., Tzeng W.P., Liebert U.G., Frey T.K. (2007). Rubella virus-induced superinfection exclusion studied in cells with persisting replicons. J. Gen. Virol..

[88] Whitaker-Dowling P., Youngner J.S. (1987). Viral interference-dominance of mutant viruses over wild-type virus in mixed infections. Microbiol. Rev..

[89] Beperet I., Irons S.L., Simón O., King L.A., Williams T., Possee R.D., López-Ferber M., Caballero P. (2014). Superinfection exclusion in alphabaculovirus infections is concomitant with actin reorganization. J. Virol..

[90] Frielle D.W., Kim P.B., Keene J.D. (1989). Inhibitory effects of vesicular stomatitis virus on cellular and influenza viral RNA metabolism and protein synthesis. Virology.

[91] Norkin L.C. (1980). Persistent infections of green monkey kidney cells initiated with temperature-sensitive mutants of simian virus 40. Virology.

[92] Norkin L.C. (1976). Rhesus monkeys kidney cells persistently infected with Simian Virus 40: Production of defective interfering virus and acquisition of the transformed phenotype. Infect. Immun..

[93] Ahmed R., Chakraborty P.R., Fields B.N. (1980). Genetic variation during lytic reovirus infection: High-passage stocks of wild-type reovirus contain temperature-sensitive mutants. J. Virol..

[94] Elliott R.M., Wilkie M.L. (1986). Persistent infection of Aedes albopictus C6/36 cells by Bunyamwera virus. Virology.

[95] Kowal K.J., Stollar V. (1980). Differential sensitivity of infectious and defective-interfering particles of Sindbis virus to ultraviolet irradiation. Virology.

[96] Peleg J., Stollar V. (1974). Homologous interference in Aedes aegypti cell cultures infected with Sindbis virus. Arch. Fur Die Gesamte Virusforsch..

[97] Shenk T.E., Koshelnyk K.A., Stollar V. (1974). Temperature-sensitive virus from Aedes albopictus cells chronically infected with Sindbis virus. J. Virol..

[98] Ju G., Birrer M., Udem S., Bloom B.R. (1980). Complementation analysis of measles virus mutants isolated from persistently infected lymphoblastoid cell lines. J. Virol..

[99] Frielle D.W., Huang D.D., Youngner J.S. (1984). Persistent infection with influenza A virus: Evolution of virus mutants. Virology.

[100] Sivaram A., Barde P.V., Gokhale M.D., Singh D.K., Mourya D.T. (2010). Evidence of co-infection of chikungunya and densonucleosis viruses in C6/36 cell lines and laboratory infected *Aedes aegypti* (L.) mosquitoes. Parasites Vectors.

[101] Wei W., Shao D., Huang X., Li J., Chen H., Zhang Q., Zhang J. (2006). The pathogenicity of mosquito densovirus (C6/36DNV) and its interaction with dengue virus type II in Aedes albopictus. Am. J. Trop. Med. Hyg..

[102] Burivong P., Pattanakitsakul S.-N., Thongrungkiat S., Malasit P., Flegel T.W. (2004). Markedly reduced severity of Dengue virus infection in mosquito cell cultures persistently infected with Aedes albopictus densovirus (AalDNV). Virology.

[103] Dasgupta R., Selling B., Rueckert R. (1994). Flock house virus: A simple model for studying persistent infection in cultured Drosophila cells. Arch. Virology. Suppl..

[104] Rechavi O., Minevich G., Hobert O. (2011). Transgenerational inheritance of an acquired small RNA-based antiviral response in C. elegans. Cell.

[105] Dasgupta R., Free H.M., Zietlow S.L., Paskewitz S.M., Aksoy S., Shi L., Fuchs J., Hu C., Christensen B.M. (2007). Replication of flock house virus in three genera of medically important insects. J. Med. Entomol..

[106] Goic B., Vodovar N., Mondotte J.A., Monot C., Frangeul L., Blanc H., Gausson V., Vera-Otarola J., Cristofari G., Saleh M.C. (2013). RNA-mediated interference and reverse transcription control the persistence of RNA viruses in the insect model Drosophila. Nat. Immunol..

[107] Whitaker-Dowling P., Youngner J.S. (1983). Vaccinia rescue of VSV from interferon-induced resistance: Reversal of translation block and inhibition of protein kinase activity. Virology.

[108] Cheng X.M., Uchida T., Xia Y.C., Umarova R., Liu C.J., Chen P.J., Gaggar A., Suri V., Mucke M.M., Vermehren J. (2020). Diminished hepatic IFN response following HCV clearance triggers HBV reactivation in coinfection. J. Clin. Investig..

[109] Hao X.Q., Li Y.C., Chen H., Chen B., Liu R.H., Wu Y.D., Xiao X.Y., Zhou P., Li S.J. (2022). Canine Circovirus Suppresses the Type I Interferon Response and Protein Expression but Promotes CPV-2 Replication. Int. J. Mol. Sci..

[110] He B., Paterson R.G., Stock N., Durbin J.E., Durbin R.K., Goodbourn S., Randall R.E., Lamb R.A. (2002). Recovery of paramyxovirus simian virus 5 with a V protein lacking the conserved cysteine-rich domain: The multifunctional V protein blocks both interferon-beta induction and interferon signaling. Virology.

[111] Wansley E.K., Grayson J.M., Parks G.D. (2003). Apoptosis induction and interferon signaling but not IFN-β promoter induction by an SV5 P/V mutant are rescued by coinfection with wild-type SV5. Virology.

[112] Du X., Zhou D., Zhou J., Xue J., Cheng Z. (2022). Marek’s Disease Virus and Reticuloendotheliosis Virus Coinfection Enhances Viral Replication and Alters Cellular Protein Profiles. Front. Vet. Sci..

[113] Li L., Zhuang P.P., Cheng Z.Q., Yang J., Bi J.M., Wang G.H. (2020). Avian leukosis virus subgroup J and reticuloendotheliosis virus coinfection induced TRIM62 regulation of the actin cytoskeleton. J. Vet. Sci..

[114] McCall L.I., Siqueira-Neto J.L., McKerrow J.H. (2016). Location, Location, Location: Five Facts about Tissue Tropism and Pathogenesis. PLoS Pathog..

[115] Ure A.E., Forslund O. (2014). Characterization of Human Papillomavirus Type 154 and Tissue Tropism of Gammapapillomaviruses. PLoS ONE.

[116] Flegel T.W. (2007). Update on viral accommodation, a model for host-viral interaction in shrimp and other arthropods. Dev. Comp. Immunol..

[117] Flegel T.W. (2012). Historic emergence, impact and current status of shrimp pathogens in Asia. J. Invertebr. Pathol..

[118] Johansson M.W., Söderhäll K. (1996). The prophenoloxidase activating system and associated proteins in invertebrates. Prog. Mol. Subcell. Biol..

[119] Namikoshi A., Wu J.L., Yamashita T., Nishizawa T., Nishioka T., Arimoto M., Muroga K. (2004). Vaccination trials with Penaeus japonicus to induce resistance to white spot syndrome virus. Aquaculture.

[120] Venegas C.A., Nonaka L., Mushiake K., Nishizawa T., Murog K. (2000). Quasi-immune response of Penaeus japonicus to penaeid rod-shaped DNA virus (PRDV). Dis. Aquat. Org..

[121] Khanobdee K., Soowannayan C., Flegel T.W., Ubol S., Withyachumnarnkul B. (2002). Evidence for apoptosis correlated with mortality in the giant black tiger shrimp Penaeus monodon infected with yellow head virus. Dis. Aquat. Org..

[122] Sahtout A.H., Hassan M.D., Shariff M. (2001). DNA fragmentation, an indicator of apoptosis, in cultured black tiger shrimp Penaeus monodon infected with white spot syndrome virus (WSSV). Dis. Aquat. Org..

[123] Wongprasert K., Khanobdee K., Glunukarn S.S., Meeratana P., Withyachumnarnkul B. (2003). Time-course and levels of apoptosis in various tissues of black tiger shrimp Penaeus monodon infected with white-spot syndrome virus. Dis. Aquat. Org..

[124] Vasilakis N., Shell E.J., Fokam E.B., Mason P.W., Hanley K.A., Estes D.M., Weaver S.C. (2007). Potential of ancestral sylvatic dengue-2 viruses to re-emerge. Virology.

[125] Rudnick A., Chan Y.C. (1965). Dengue Type 2 Virus in Naturally Infected Aedes albopictus Mosquitoes in Singapore. Science.

[126] Vasilakis N., Holmes E.C., Fokam E.B., Faye O., Diallo M., Sall A.A., Weaver S.C. (2007). Evolutionary processes among sylvatic dengue type 2 viruses. J. Virol..

[127] Linde A., Rotzén-Östlund M., Zweygberg-Wirgart B., Rubinova S., Brytting M. (2009). Does viral interference affect spread of influenza?. Eurosurveillance.

[128] Magalhaes T., Robison A., Young M.C., Black W.C., Foy B.D., Ebel G.D., Ruckert C. (2018). Sequential Infection of Aedes aegypti Mosquitoes with Chikungunya Virus and Zika Virus Enhances Early Zika Virus Transmission. Insects.

[129] Wernery U., Kaaden O.R. (2004). Foot-and-mouth disease in camelids: A review. Vet. J..

[130] Kumar N., Barua S., Riyesh T., Chaubey K.K., Rawat K.D., Khandelwal N., Mishra A.K., Sharma N., Chandel S.S., Sharma S. (2016). Complexities in Isolation and Purification of Multiple Viruses from Mixed Viral Infections: Viral Interference, Persistence and Exclusion. PLoS ONE.

[131] Goka E.A., Vallely P.J., Mutton K.J., Klapper P.E. (2015). Single, dual and multiple respiratory virus infections and risk of hospitalization and mortality. Epidemiol. Infect..

[132] Goka E., Vallely P., Mutton K., Klapper P. (2013). Influenza A viruses dual and multiple infections with other respiratory viruses and risk of hospitalisation and mortality. Influenza Other Respir Viruses.

[133] Tang M.B., Yu C.P., Chen S.C., Chen C.H. (2014). Co-Infection of Adenovirus, Norovirus and Torque Teno Virus in Stools of Patients with Acute Gastroenteritis. Southeast Asian J. Trop. Med. Public Health.

[134] Amin J., Law M.G., Bartlett M., Kaldor J.M., Dore G.J. (2006). Causes of death after diagnosis of hepatitis B or hepatitis C infection: A large community-based linkage study. Lancet.

[135] Liang L., He C., Lei M., Li S., Hao Y., Zhu H., Duan Q. (2005). Pathology of guinea pigs experimentally infected with a novel reovirus and coronavirus isolated from SARS patients. DNA Cell Biol..

[136] Calixto R., Oliveira G., Lima M., Andrade A.C., Trindade G.D., De Oliveira D.B., Kroon E.G. (2018). A Model to Detect Autochthonous Group 1 and 2 Brazilian Vaccinia virus Coinfections: Development of a qPCR Tool for Diagnosis and Pathogenesis Studies. Viruses.

[137] Guan Z., Chen C., Li Y., Yan D., Zhang X., Jiang D., Yang S., Li L. (2021). Impact of Coinfection With SARS-CoV-2 and Influenza on Disease Severity: A Systematic Review and Meta-Analysis. Front. Public Heal..

[138] Xiang T.D., Zheng X. (2021). Interaction between hepatitis B virus and SARS-CoV-2 infections. World J. Gastroenterol..

[139] Martinez-Roig A., Salvado M., Caballero-Rabasco M.A., Sanchez-Buenavida A., Lopez-Segura N., Bonet-Alcaina M. (2015). Viral Coinfection in Childhood Respiratory Tract Infections. Arch. Bronconeumol..

[140] Zeremski M., Martinez A.D., Talal A.H. (2014). Editorial Commentary: Management of Hepatitis C Virus in HIV-Infected Patients in the Era of Direct-Acting Antivirals. Clin. Infect. Dis..

[141] Mercado-Reyes M., Acosta-Reyes J., Navarro-Lechuga E., Corchuelo S., Rico A., Parra E., Tolosa N., Pardo L., González M., Martìn-Rodriguez-Hernández J. (2019). Dengue, chikungunya and zika virus coinfection: Results of the national surveillance during the zika epidemic in Colombia. Epidemiol. Infect..

[142] Mahajan S., Sharma G.K., Subramaniam S., Biswal J.K., Pattnaik B. (2021). Selective isolation of foot-and-mouth disease virus from coinfected samples containing more than one serotype. Braz. J. Microbiol..

[143] Scotta M.C., Chakr V.C., de Moura A., Becker R.G., de Souza A.P., Jones M.H., Pinto L.A., Sarria E.E., Pitrez P.M., Stein R.T. (2016). Respiratory viral coinfection and disease severity in children: A systematic review and meta-analysis. J. Clin. Virol..

[144] Semple M.G., Cowell A., Dove W., Greensill J., McNamara P.S., Halfhide C., Shears P., Smyth R.L., Hart C.A. (2005). Dual infection of infants by human metapneumovirus and human respiratory syncytial virus is strongly associated with severe bronchiolitis. J. Infect. Dis..

[145] Simon-Loriere E., Faye O., Prot M., Casademont I., Fall G., Fernandez-Garcia M.D., Diagne M.M., Kipela J.M., Fall I.S., Holmes E.C. (2017). Autochthonous Japanese Encephalitis with Yellow Fever Coinfection in Africa. New Engl. J. Med..

[146] Salassa B., Daziano E., Bonino F., Lavarini C., Smedile A., Chiaberge E., Rosina F., Brunetto M.R., Pessione E., Spezia C. (1991). Serological diagnosis of hepatitis B and delta virus (HBV/HDV) coinfection. J. Hepatol..

[147] Masyeni S., Santoso M.S., Widyaningsih P.D., Asmara D.G.W., Nainu F., Harapan H., Sasmono R.T. (2021). Serological cross-reaction and coinfection of dengue and COVID-19 in Asia: Experience from Indonesia. Int. J. Infect. Dis..

[148] Costa-Hurtado M., Afonso C.L., Miller P.J., Shepherd E., DeJesus E., Smith D., Pantin-Jackwood M.J. (2016). Effect of Infection with a Mesogenic Strain of Newcastle Disease Virus on Infection with Highly Pathogenic Avian Influenza Virus in Chickens. Avian. Dis..

[149] Bara J.J., Muturi E.J. (2014). Effect of mixed infections of Sindbis and La Crosse viruses on replication of each virus in vitro. Acta Trop..

[150] Li X., Zhang K.R., Pei Y., Xue J., Ruan S.F., Zhang G.Z. (2020). Development and Application of an MRT-qPCR Assay for Detecting Coinfection of Six Vertically Transmitted or Immunosuppressive Avian Viruses. Front. Microbiol..

[151] Xu Z.X., Peng Y., Yang M.H., Li X.H., Wang J., Zou R.R., Liang J.H., Fang S.S., Liu Y.X., Yang Y. (2022). Simultaneous detection of Zika, chikungunya, dengue, yellow fever, West Nile, and Japanese encephalitis viruses by a two-tube multiplex real-time RT-PCR assay. J. Med. Virol..

[152] Sanchez-Ponce Y., Varela-Fascinetto G., Romo-Vazquez J.C., Lopez-Martinez B., Sanchez-Huerta J.L., Parra-Ortega I., Fuentes-Panana E.M., Morales-Sanchez A. (2018). Simultaneous Detection of Beta and Gamma Human Herpesviruses by Multiplex qPCR Reveals Simple Infection and Coinfection Episodes Increasing Risk for Graft Rejection in Solid Organ Transplantation. Viruses.

[153] Leibovitch E.C., Brunetto G.S., Caruso B., Fenton K., Ohayon J., Reich D.S., Jacobson S. (2014). Coinfection of Human Herpesviruses 6A (HHV-6A) and HHV-6B as Demonstrated by Novel Digital Droplet PCR Assay. PLoS ONE.

[154] Xi D., Luo X., Ning Q. (2007). Detection of HBV and HCV coinfection by TEM with Au nanoparticle gene probes. J. Huazhong Univ. Sci. Technol..

[155] Fayyadh T.K., Ma F.Y., Qin C., Zhang X.W., Li W., Zhang X.E., Zhang Z.P., Cui Z.Q. (2017). Simultaneous detection of multiple viruses in their co-infected cells using multicolour imaging with self-assembled quantum dot probes. Microchim. Acta.

[156] Srisomwat C., Yakoh A., Avihingsanon A., Chuaypen N., Tangkijvanich P., Vilaivan T., Chailapakul O. (2022). An alternative label-free DNA sensor based on the alternating-current electroluminescent device for simultaneous detection of human immunodeficiency virus and hepatitis C co-infection. Biosens. Bioelectron..

[157] Garcia-Hernandez M.-E., Trujillo-Ortega M.-E., Alcaraz-Estrada S.-L., Lozano-Aguirre-Beltran L., Sandoval-Jaime C., Taboada-Ramirez B.I., Sarmiento-Silva R.-E. (2021). Molecular Detection and Characterization of Porcine Epidemic Diarrhea Virus and Porcine Aichivirus C Coinfection in Mexico. Viruses.

[158] Bialasiewicz S., McVernon J., Nolan T., Lambert S.B., Zhao G., Wang D., Nissen M.D., Sloots T.P. (2014). Detection of a divergent Parainfluenza 4 virus in an adult patient with influenza like illness using next-generation sequencing. BMC Infect. Dis..

[159] Visser M., Bester R., Burger J.T., Maree H.J. (2016). Next-generation sequencing for virus detection: Covering all the bases. Virol. J..

[160] van Engelenburg F.A., Terpstra F.G., Schuitemaker H., Moorer W.R. (2002). The virucidal spectrum of a high concentration alcohol mixture. J. Hosp. Infect..

[161] Leland Diane S., Ginocchio Christine C. (2007). Role of Cell Culture for Virus Detection in the Age of Technology. Clin. Microbiol. Rev..

[162] Beperet I., Simón O., López-Ferber M., Lent J.v., Williams T., Caballero P., Johnson K.N. (2021). Mixtures of Insect-Pathogenic Viruses in a Single Virion: Towards the Development of Custom-Designed Insecticides. Appl. Environ. Microbiol..

[163] Chang S.-F., Su C.-L., Shu P.-Y., Yang C.-F., Liao T.-L., Cheng C.-H., Hu H.-C., Huang J.-H. (2010). Concurrent Isolation of Chikungunya Virus and Dengue Virus from a Patient with Coinfection Resulting from a Trip to Singapore. J. Clin. Microbiol..

[164] Davidson I., Nagar S., Haddas R., Ben-Shabat M., Golender N., Lapin E., Altory A., Simanov L., Ribshtein I., Panshin A. (2010). Avian Influenza Virus H9N2 Survival at Different Temperatures and pHs. Avian Dis..

[165] Lee K.W., Chi S.C., Cheng T.M. (2002). Interference of the life cycle of fish nodavirus with fish retrovirus. J. Gen. Virol..

[166] Harada Y., Takahashi H., Trusheim H., Roth B., Mizuta K., Hirata-Saito A., Ogane T., Odagiri T., Tashiro M., Yamamoto N. (2020). Comparison of suspension MDCK cells, adherent MDCK cells, and LLC-MK2 cells for selective isolation of influenza viruses to be used as vaccine seeds. Influenza Other Respir. Viruses.

[167] Zhang W., Kataoka M., Doan H.Y., Wu F.-T., Takeda N., Muramatsu M., Li T.-C. (2021). Isolation and Characterization of Hepatitis E Virus Subtype 4b Using a PLC/PRF/5 Cell-Derived Cell Line Resistant to Porcine Sapelovirus Infection. Jpn. J. Infect. Dis..

[168] Kim J., Chae C. (2004). A comparison of virus isolation, polymerase chain reaction, immunohistochemistry, and in situ hybridization for the detection of porcine circovirus 2 and porcine parvovirus in experimentally and naturally coinfected pigs. J. Vet. Diagn. Investig..

[169] Dormitorio T.V., Giambrone J.J. (2010). Limiting dilution studies to detect avian influenza viruses from questionable allantoic fluid samples. J. Dairy Sci..

[170] El Zowalatys M.E., Chander Y., Redig P.T., El Latif H.K.A., El Sayed M.A., Goyal S.M. (2011). Selective isolation of Avian influenza virus (AIV) from cloacal samples containing AIV and Newcastle disease virus. J. Vet. Diagn. Investig..

[171] Mi S.J., Guo S.B., Xing C.N., Xiao C.T., He B., Wu B., Xia X.Z., Tu C.C., Gong W.J. (2021). Isolation and Characterization of Porcine Astrovirus 5 from a Classical Swine Fever Virus-Infected Specimen. J. Virol..

[172] Neighbor N.K., Newberry L.A., Bayyari G.R., Skeeles J.K., Beasley J.N., McNew R.W. (1994). The effect of microaerosolized hydrogen peroxide on bacterial and viral poultry pathogens. Poult. Sci..

[173] Brown J.D., Goekjian G., Poulson R., Valeika S., Stallknecht D.E. (2009). Avian influenza virus in water: Infectivity is dependent on pH, salinity and temperature. Vet. Microbiol..

[174] Moses H.E., Brandly C.A., Jones E.E. (1947). The pH Stability of Viruses of Newcastle Disease and Fowl Plague. Science.

[175] Hematian A., Sadeghifard N., Mohebi R., Taherikalani M., Nasrolahi A., Amraei M., Ghafourian S. (2016). Traditional and Modern Cell Culture in Virus Diagnosis. Osong Public Health Res. Perspect..

[176] Waner J.L. (1994). Mixed viral infections: Detection and management. Clin. Microbiol. Rev..

[177] Brumback B.G., Wade C.D. (1994). Simultaneous culture for adenovirus, cytomegalovirus, and herpes simplex virus in same shell vial by using three-color fluorescence. J. Clin. Microbiol..

[178] Weinberg A., Brewster L., Clark J., Simoes E. (2004). Evaluation of R-Mix shell vials for the diagnosis of viral respiratory tract infections. J. Clin. Virol..

[179] Lim G., Park T.S., Suh J.T., Lee H.J. (2010). Comparison of R-mix virus culture and multiplex reverse transcriptase-PCR for the rapid detection of respiratory viruses. Korean J. Lab. Med..

[180] Barenfanger J., Drake C., Mueller T., Troutt T., O’Brien J., Guttman K. (2001). R-Mix cells are faster, at least as sensitive and marginally more costly than conventional cell lines for the detection of respiratory viruses. J. Clin. Virol..

[181] Dunn J.J., Woolstenhulme R.D., Langer J., Carroll K.C. (2004). Sensitivity of respiratory virus culture when screening with R-mix fresh cells. J. Clin. Microbiol..

[182] St George K., Patel N.M., Hartwig R.A., Scholl D.R., Jollick J.A., Kauffmann L.M., Evans M.R., Rinaldo C.R. (2002). Rapid and sensitive detection of respiratory virus infections for directed antiviral treatment using R-Mix cultures. J. Clin. Virol..

[183] Gillim-Ross L., Taylor J., Scholl D.R., Ridenour J., Masters P.S., Wentworth D.E. (2004). Discovery of novel human and animal cells infected by the severe acute respiratory syndrome coronavirus by replication-specific multiplex reverse transcription-PCR. J. Clin. Microbiol..

[184] Kim D.K., Poudel B. (2013). Tools to detect influenza virus. Yonsei Med. J..

[185] Yuen K.Y., Chan P.K., Peiris M., Tsang D.N., Que T.L., Shortridge K.F., Cheung P.T., To W.K., Ho E.T., Sung R. (1998). Clinical features and rapid viral diagnosis of human disease associated with avian influenza A H5N1 virus. Lancet.

[186] Choi W.S., Noh J.Y., Baek J.H., Seo Y.B., Lee J., Song J.Y., Park D.W., Lee J.S., Cheong H.J., Kim W.J. (2015). Suboptimal effectiveness of the 2011-2012 seasonal influenza vaccine in adult Korean populations. PLoS ONE.

[187] Schmutzhard J., Merete Riedel H., Zweygberg Wirgart B., Grillner L. (2004). Detection of herpes simplex virus type 1, herpes simplex virus type 2 and varicella-zoster virus in skin lesions. Comparison of real-time PCR, nested PCR and virus isolation. J. Clin. Virol..

[188] Huang Y.T., Hite S., Duane V., Yan H. (2002). CV-1 and MRC-5 mixed cells for simultaneous detection of herpes simplex viruses and varicella zoster virus in skin lesions. J. Clin. Virol..

[189] Woreta T.A., Chalasani N. (2021). Fatty Liver Disease in Human Immunodeficiency Virus-Hepatitis B Virus Coinfection: A Cause for Concern COMMENT. Clin. Infect. Dis..

[190] Kim Z., Lee J.H. (2021). Coinfection with severe acute respiratory syndrome coronavirus-2 and other respiratory viruses at a tertiary hospital in Korea. J Clin Lab Anal.

[191] Zhong P.P., Zhang H.L., Chen X.F., Lv F.F. (2019). Clinical characteristics of the lower respiratory tract infection caused by a single infection or coinfection of the human parainfluenza virus in children. J. Med. Virol..

[192] Mavilia M.G., Wu G.Y. (2018). HBV-HCV Coinfection: Viral Interactions, Management, and Viral Reactivation. J. Clin. Transl. Hepatol..

[193] Almajhdi F.N., Ali G. (2013). Report on Influenza A and B Viruses: Their Coinfection in a Saudi Leukemia Patient. Biomed Res. Int..

[194] Yapali S., Bayrakci B., Gunsar F., Ersoz G., Karasu Z., Akarca U.S. (2011). Delta virus coinfection does not increase, but hcv coinfection increase the hbsag loss, in chronic hbv infection. J. Hepatol..

[195] Kim E.H., Nguyen T.Q., Casel M.A.B., Rollon R., Kim S.M., Kim Y.I., Yu K.M., Jang S.G., Yang J., Poo H. (2022). Coinfection with SARS-CoV-2 and Influenza A Virus Increases Disease Severity and Impairs Neutralizing Antibody and CD4(+) T Cell Responses. J. Virol..

[196] Pires C.A.A., Quaresma J.A.S., Aarao T.L.D., de Souza J.R., Macedo G.M.M., Neto F., Xavier M.B. (2017). Expression of interleukin-1 beta and interleukin-6 in leprosy reactions in patients with human immunodeficiency virus coinfection. Acta Trop..

[197] Kanthong N., Khemnu N., Pattanakitsakul S.-N., Malasit P., Flegel T.W. (2010). Persistent, triple-virus co-infections in mosquito cells. BMC Microbiol..

[198] Cook J.K., Huggins M.B., Orbell S.J., Mawditt K., Cavanagh D. (2001). Infectious bronchitis virus vaccine interferes with the replication of avian pneumovirus vaccine in domestic fowl. Avian Pathol. J. WVPA.

[199] Gelb J., Ladman B.S., Licata M.J., Shapiro M.H., Campion L.R. (2007). Evaluating viral interference between infectious bronchitis virus and Newcastle disease virus vaccine strains using quantitative reverse transcription-polymerase chain reaction. Avian Dis..

[200] Pantin-Jackwood M.J., Costa-Hurtado M., Miller P.J., Afonso C.L., Spackman E., Kapczynski D.R., Shepherd E., Smith D., Swayne D.E. (2015). Experimental co-infections of domestic ducks with a virulent Newcastle disease virus and low or highly pathogenic avian influenza viruses. Vet. Microbiol..

[201] Sloutskin A., Yee M.B., Kinchington P.R., Goldstein R.S. (2014). Varicella-zoster virus and herpes simplex virus 1 can infect and replicate in the same neurons whether co- or superinfected. J. Virol..

[202] White P.A., Li Z., Zhai X., Marinos G., Rawlinson W.D. (2000). Mixed viral infection identified using heteroduplex mobility analysis (HMA). Virology.

[203] Diallo I.S., Taylor J., Gibson J., Hoad J., De Jong A., Hewitson G., Corney B.G., Rodwell B.J. (2010). Diagnosis of a naturally occurring dual infection of layer chickens with fowlpox virus and gallid herpesvirus 1 (infectious laryngotracheitis virus). Avian Pathol. J. WVPA.

[204] Otta S.K., Arulraj R., Ezhil Praveena P., Manivel R., Panigrahi A., Bhuvaneswari T., Ravichandran P., Jithendran K.P., Ponniah A.G. (2014). Association of dual viral infection with mortality of Pacific white shrimp (Litopenaeus vannamei) in culture ponds in India. VirusDisease.

[205] Vaziry A., Silim A., Bleau C., Frenette D., Lamontagne L. (2013). Dual infections with low virulent chicken infectious anaemia virus (lvCIAV) and intermediate infectious bursal disease virus (iIBDV) in young chicks increase lvCIAV in thymus and bursa while decreasing lymphocyte disorders induced by iIBDV. Avian Pathol. J. WVPA.

[206] Ge X.-Y., Wang N., Zhang W., Hu B., Li B., Zhang Y.-Z., Zhou J.-H., Luo C.-M., Yang X.-L., Wu L.-J. (2016). Coexistence of multiple coronaviruses in several bat colonies in an abandoned mineshaft. Virol. Sin..

[207] Esposito S., Zampiero A., Bianchini S., Mori A., Scala A., Tagliabue C., Sciarrabba C.S., Fossali E., Piralla A., Principi N. (2016). Epidemiology and Clinical Characteristics of Respiratory Infections Due to Adenovirus in Children Living in Milan, Italy, during 2013 and 2014. PLoS ONE.

[208] González Álvarez D.A., López Cortés L.F., Cordero E. (2016). Impact of HIV on the severity of influenza. Expert Rev. Respir. Med..

[209] Zhou N., Fan C., Liu S., Zhou J., Jin Y., Zheng X., Wang Q., Liu J., Yang H., Gu J. (2017). Cellular proteomic analysis of porcine circovirus type 2 and classical swine fever virus coinfection in porcine kidney-15 cells using isobaric tags for relative and absolute quantitation-coupled LC-MS/MS. Electrophoresis.

[210] Shinjoh M., Omoe K., Saito N., Matsuo N., Nerome K. (2000). In vitro growth profiles of respiratory syncytial virus in the presence of influenza virus. Acta Virol..

[211] Hurt A.C., Nor’e S.S., McCaw J.M., Fryer H.R., Mosse J., McLean A.R., Barr I.G. (2010). Assessing the viral fitness of oseltamivir-resistant influenza viruses in ferrets, using a competitive-mixtures model. J. Virol..

[212] Dobrescu I., Levast B., Lai K., Delgado-Ortega M., Walker S., Banman S., Townsend H., Simon G., Zhou Y., Gerdts V. (2014). In vitro and ex vivo analyses of co-infections with swine influenza and porcine reproductive and respiratory syndrome viruses. Vet. Microbiol..

[213] Rozeboom L.E., Kassira E.N. (1969). Dual infections of mosquitoes with strains of West Nile virus. J. Med. Entomol..

[214] Pesko K., Mores C.N. (2009). Effect of sequential exposure on infection and dissemination rates for West Nile and St. Louis encephalitis viruses in Culex quinquefasciatus. Vector-Borne Zoonotic Dis..

[215] Dittmar D., Castro A., Haines H. (1982). Demonstration of interference between dengue virus types in cultured mosquito cells using monoclonal antibody probes. J. Gen. Virol..

